# Genome-Wide Multiple Sclerosis Association Data and Coagulation

**DOI:** 10.3389/fneur.2019.00095

**Published:** 2019-02-14

**Authors:** Sara La Starza, Michela Ferraldeschi, Maria Chiara Buscarinu, Silvia Romano, Arianna Fornasiero, Rosella Mechelli, Renato Umeton, Giovanni Ristori, Marco Salvetti

**Affiliations:** ^1^Geriatrics, Neuroscience, Orthopaedics, Head and Neck Department, Fondazione Policlinico Universitario A. Gemelli IRCCS, Rome, Italy; ^2^Department of Neuroscience, Mental Health and Sensory Organs, Faculty of Medicine and Psychology, Centre for Experimental Neurological Therapies, S. Andrea Hospital, Sapienza University, Rome, Italy; ^3^Department of Human Science and Promotion of Quality of Life, San Raffaele Roma Open University, Rome, Italy; ^4^Department of Informatics & Analytics, Dana-Farber Cancer Institute, Boston, MA, United States; ^5^Massachusetts Institute of Technology, Cambridge, MA, United States; ^6^IRCCS Istituto Neurologico Mediterraneo (INM) Neuromed, Pozzilli, Italy

**Keywords:** multiple sclerosis, genome-wide association studies, cluster of differentiation 40, plasminogen activator, urokinase gene, connectivity analysis

## Abstract

The emerging concept of a crosstalk between hemostasis, inflammation, and immune system prompt recent works on coagulation cascade in multiple sclerosis (MS). Studies on MS pathology identified several coagulation factors since the beginning of the disease pathophysiology: fibrin deposition with breakdown of blood brain barrier, and coagulation factors within active plaques may exert pathogenic role, especially through the innate immune system. Studies on circulating coagulation factors showed complex imbalance involving several components of hemostasis cascade (thrombin, factor X, factor XII). To analyze the role of the coagulation process in connection with other pathogenic pathways, we implemented a systematic matching of genome-wide association studies (GWAS) data with an informative and unbiased network of coagulation pathways. Using MetaCore (version 6.35 build 69300, 2018) we analyzed the connectivity (i.e., direct and indirect interactions among two networks) between the network of the coagulation process and the network resulting from feeding into MetaCore the MS GWAS data. The two networks presented a remarkable over-connectivity: 958 connections vs. 561 expected by chance; *z*-score = 17.39; *p*-value < 0.00001. Moreover, genes coding for cluster of differentiation 40 (CD40) and plasminogen activator, urokinase (PLAU) shared both networks, pointed to an integral interplay between coagulation cascade and main pathogenic immune effectors. In fact, CD40 pathways is especially operative in B cells, that are currently a major therapeutic target in MS field. The potential interaction of PLAU with a signal of paramount importance for B cell pathogenicity, such as CD40, suggest new lines of research and pave the way to implement new therapeutic targets.

## Introduction

Recent studies focused on the role of coagulation cascade in neuroinflammation and neurodegenerative disease, considering new suggestions on a crosstalk between hemostasis, inflammation and immune system (1). The majority of these studies regarded multiple sclerosis (MS), but others demonstrated a dysregulation of several proteins of the coagulation cascade in many other central nervous system (CNS) diseases: traumatic brain and spinal cord injury, Parkinson disease, amyotrophic lateral sclerosis, Huntington disease and Alzheimer dementia (2–6).

A recent review discussed the role of fibrinogen in some neurological diseases, with an emphasis on the cellular targets and the fibrinogen-induced signal transduction pathways in the CNS: fibrinogen has a pleiotropic role in the activation of inflammation and pathologies that share, as common change, the increased blood-brain barrier (BBB) permeability. This produces the extravasation of plasma proteins that are undetectable in a healthy CNS, but abundantly deposited in many neurological conditions, whereby they mediate both pathological inflammation and tissue repair (7).

In MS the BBB breakdown and activation of the innate immune system appears to be an early event in the diseases development, that may precede the clinical onset. Different studies showed that fibrin deposition is a leading feature of MS pathology and it is presents all over the disease course (7). Fibrinogen can directly activate microglia cells *in vitro* and increase their phagocytic ability by binding to the integrin receptor CD11b/CD18, which is specifically expressed in the CNS (8). Participation of the coagulation cascade to the neuropathology of MS was strongly suggested by a proteomic analysis on laser-micro dissected, post-mortem brain lesions. Comparative proteomic profiles identified tissue factor and protein C inhibitor within chronic active plaque samples. *In vivo* experiments with antagonists of the coagulation factors identified (hirudin or recombinant activated protein C) were capable of ameliorating animal models of MS and suppressing pathogenic immune effectors, confirming the impact of dysregulated coagulation factors on demyelinating processes and suggesting potential therapeutic targets (9).

Another approach focused on the study of circulating coagulation factors, as possible biomarkers and targets of treatment tactics in MS pathogenic process. Gobel et al. (10) studied different neurological diseases (all the forms of MS, neuro myelitis optica spectrum disorders, other inflammatory neurological diseases, and non-inflammatory neurological conditions) compared to healthy status. The plasma levels of different coagulation proteins measured and the results demonstrated significantly higher levels of prothrombin and factor X in MS patients, without significant changes in the other conditions. Thrombin produces different inflammatory responses, including platelet activation, vasodilatation, leukocyte attraction, production of cytokine, and chemokine (IL-1, IL-6, TNFα) (11). These effects in CNS are also dependent on thrombin concentration: at low-to-moderate concentrations, it protects hippocampal neurons and astrocytes from insults, while at higher concentrations thrombin induces cell death (12, 13). Another coagulation factor that proved to be somehow involved in MS pathogenic process was factor XII (FXII). Increased FXII levels and reduced function within the intrinsic coagulation pathway were evident in people with MS (14); Gobel et al. found high levels of FXII activity in the plasma of MS patients during relapse, and immune activating effects mediated by interactions between FXII and dendritic cells in a CD87-dependent manner (15).

The above studies [with the prominent exception of the proteomic analysis by Han et al. (9)] were planned with a hypothesis-driven approach focusing on single factors of coagulation cascade. The coming of genome-wide association studies (GWAS) data would allow unbiased approaches capable of disclosing a more extensive landscape of coagulation process involvement in MS pathogenesis. GWAS results are derived from population-based association studies, comparing disease cases and controls for common genetic variants, that have variable frequencies in the general population. Each common variants (signaled by a single nucleotide polymorphism) explain a small fraction of the risk/protection in a population. The overall MS genetic risk is multifaceted: many common variants of small effect spread throughout the genome, loci of stronger effects lying in the human leukocyte antigen (HLA) haplotype, that had been associated to disease risk since eighties, as well as recently described low-frequency and rare-coding variants all contribute to the complex genetic architecture of MS (16).

## GWAS Studies And Coagulation

GWAS studies encompassing the last decade have identified more than 200 MS-associated loci across the human genome (17). Technological advances, adequate increase of sample size, and improved statistical approaches have all contributed to a substantial progress in the definition of the complex genetic architecture of MS. This prompted a significant extension of the view on MS genetics, that was essentially limited to the role of human histocompatibility haplo types until 15 years ago. At least two challenges remain: (i) the definition of a comprehensive etiological model, with the need of better understanding both the plausibly causal effects in altering disease risk for many of the susceptibility gene regions identified, and the impact of non-genetic factors, as demonstrated, among others, by twin studies (18, 19); (ii**)** the clinical translation of genomic data, that may exploit the relevance of pathogenic pathways, for which therapeutics is already available in clinical practice, or may drive the discovery of new druggable targets.

One potentially informative approach to deal with these issues includes bioinformatics attempts capable of extracting from GWAS data the biological consequences and the functional implications of individual disease-associated variants. Our group implemented analyses aimed at clarifying the interplay between diseases-associated genomic regions and presumed causal environmental factors (20–22). Another bioinformatics reworking allows to explore the reciprocal interactions of pathways resulting from GWAS data, to disclose unknown networks and to focus on previously under estimated pathways in MS etiology.

**Figure 1:**
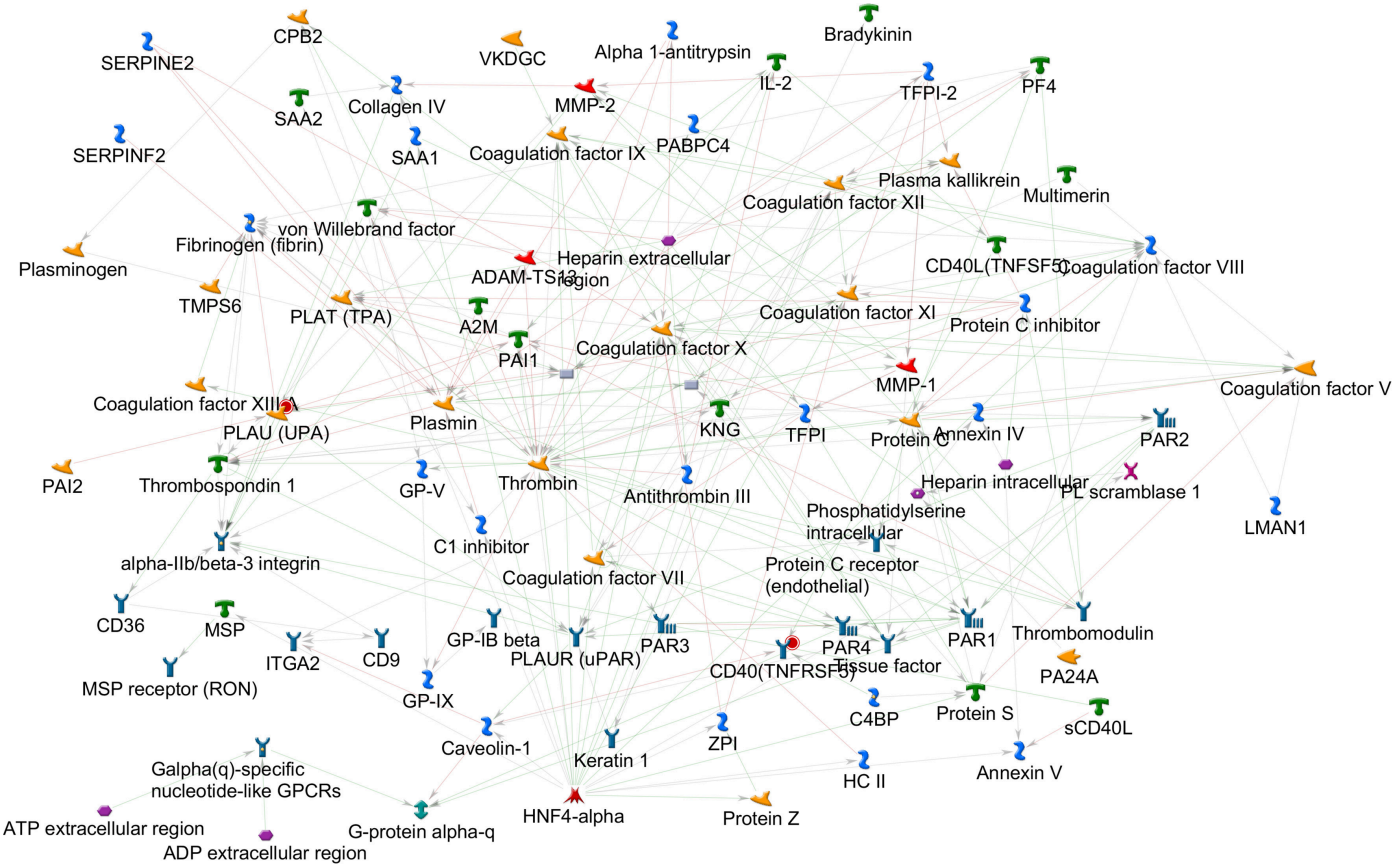
Coagulation process map, as available in MetaCore 2018. The elements on this map, together with their interactors, were used for the connectivity analysis.

**Figure 2:**
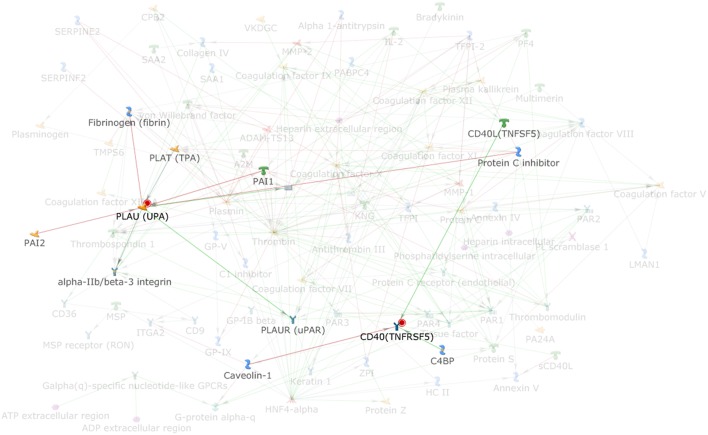
Highlight of the elements (PLAU and CD40) that are shared among the coagulation process map and the MS GWAS gene list; PLAU and CD40 interactors are also highlighted.

By applying the latter approach we used bioinformatics tools to analyze the role of the coagulation cascade in connection with other biological pathways contributing to the complex disease pathogenesis. Using MetaCore (version 6.35 build 69300, 2018) we analyzed the connectivity (i.e., direct and indirect interactions among two networks) between the network of the coagulation process (a standard map in MetaCore, presented in Figure 1, that includes 94 components) and the network resulting from feeding into MetaCore the MS GWAS data. In particular we considered genes that were reported in 19 MS GWAS studies (23–41) filed in the GWAS Catalog (https://www.ebi.ac.uk/gwas); such list (Supplementary Table 1) contains 398 genes, that were either reported as associated to MS in the aforementioned studies, or that were originally reported as hits on non-well specified regions, later mapped to better characterized regions and genes. The connectivity analysis in MetaCore takes place in two steps: first, the genes that are shared by the two networks (i.e., elements that appear in both the coagulation process network and the MS GWAS network) are identified; second, every element in each network is enriched with its interactors. A statistics is then computed counting how many interactions are observed among the two enriched networks, comparing this number to what would be expected by chance. MetaCore connectivity analysis showed the following results: the coagulation process network and the MS GWAS network presented a remarkable over-connectivity, showing 958 connections (561 were expected by chance) that lead to *z*-score of 17.39 and *p*-value < 0.00001; genes coding for cluster of differentiation 40 (CD40) and plasminogen activator, urokinase (PLAU) appeared both in the coagulation process network and the MS GWAS network (Figure 2).

These analyses on one hand confirm that the coagulation cascade may have an impact on MS development, as already reported (see above), on the other hand fail to detect main coagulation components previously indicated by experimental studies. This limitation may pertain the analyses based on GWAS studies in general, which incorporate huge number of gene variants and several levels of possible functional complexities. Specifically, PLAU pathway has already been scrutinized for its role in the activation of matrix metallopeptidase9, that has in turn been associated with BBB breakdown, a crucial event in MS development (42). However, the network sharing by PLAU and CD40 pathways, resulting from our analysis, points to a more integral interplay between coagulation cascade and immune effectors, that are currently the main focus of research on MS etiopathogenesis and therapy. CD40 pathways is especially operative in B cells, being the typical signal mediating help by T cells (through CD40 ligand) on cognate B lymphocytes for antibody production and other important functions, such as antigen presenting cells and cells modulating the immune response. Recent studies show indeed that MS-associated genetic variants alter the expression of co-stimulatory molecule, including CD40 in B cells, as well as the level of steering cytokines such as interleukin-10, which is considered to have an immunoregulatory function downstream of CD40 (43). Moreover, the CD40-CD40 ligand dyadis intensively investigated for its essential role in the development of MS, with the aim of targeting it therapeutically and antagonize neuroinflammation (44).

The role of CD40 pathway in MS development refers to the more general topic of the role of B cells in neuroinflammation. Our recent works suggest that B lymphocytes, in an activated and pro-survival status, contribute to MS development with functions other than antibody-production (45). Indeed, B lymphocytes are professional antigen-presenting cells for autoreactive T cells (43, 46), as well as potent producers of steering cytokines and other immune effectors influencing both pathogenic (lymphotoxin, tumor necrosis factor, granulocyte macrophage-colony stimulating factor, and metallo-peptidases) and protective (interleukin 10) milieus in neuroinflammation (47–49). Accordingly, CD20-targeted monoclonal antibodies, that deplete B cells in their earlier stages of development, turned out to be highly and consistently effective in tackling the disease development (50, 51). Hence, the finding that PLAU pathway may potentially interact with a signal of paramount importance for B cell pathogenicity, such as CD40, may open new perspectives for translational research. Along this line, the protease activity of microglial cells activated by urokinase plasminogen activator coupled with its receptor seems very important for their pathogenic role in MS (52) and, notably, this pathogenic role is increasingly recognized also in a very recent GWAS study on MS.

## Conclusion

The case of the relationship between coagulation pathway and MS molecular model may teach us how fruitful a bioinformatics reworking of GWAS data may be. In particular bioinformatics approaches that match GWAS data with other biological repositories of unbiased comprehensive records may shed light on the functional relevance of common diseases-associated single nucleotide polymorphism: each genetic variant is often located in regulatory genomic regions, and may be active in different ways in diverse tissues, making it very difficult to encompass a detailed understanding of the underpinning pathobiology.

Future works based on connectivity analyses may inform a number of questions that are still open in the context of MS heritability: the degree of epistasis and interaction with non-genetic causative factors; the existence of genetic interactors determining disease forms, clinical course, and response to diseases modifying therapies; the predictivity of endophenotypes, in particular the imaging data, that often segregate on a familiar basis. Moreover, the discovery of “clinically actionable genes” may represent a timely task in the current landscape of MS therapeutics.

Many new diseases modifying therapies, already available in clinical practice, show superior effectiveness compared to the treatments that were in place only a decade ago. The “cost” is the safety profile, being at least suboptimal. Approaches based on drugs targeting PLAU system, that have successfully been used to ameliorate CNS inflammation (53, 54), may be potential resources, with good therapeutic index and synergic action with currently available immune-modulators, potentially to be exploited in combination schemes.

## Author Contributions

All authors listed have made a substantial, direct and intellectual contribution to the work, and approved it for publication.

### Conflict of Interest Statement

The authors declare that the research was conducted in the absence of any commercial or financial relationships that could be construed as a potential conflict of interest.

## Supplementary Material

Supplementary Table 1 List of genes that were gathered from the GWAS Catalog as associated with MS and that were used for this work.

## Abbreviations

GWAS: genome-wide association studies
PLAU: plasminogen activator, urokinase.
